# Waist circumference as the predominant contributor to the micro-inflammatory response in the metabolic syndrome: a cross sectional study

**DOI:** 10.1186/1476-9255-7-35

**Published:** 2010-07-26

**Authors:** Ori Rogowski, Itzhak Shapira, Orit Kliuk-Ben Bassat, Tamar Chundadze, Talya Finn, Shlomo Berliner, Arie Steinvil

**Affiliations:** 1 Departments of Internal Medicine “D” and “E”, Tel-Aviv Sourasky Medical Center, affiliated to the Sackler Faculty of Medicine Tel-Aviv University, 6 Weizman Street, Tel Aviv 64239, Israel; 2 The Institute for Special Medical Examinations (MALRAM), Tel Aviv Sourasky Medical Center, 6 Weizman street, Tel Aviv 64239, Israel

## Abstract

**Background:**

The metabolic syndrome (MetS) is associated with the presence of low grade inflammation. Our aim was to analyze the inter-relations between each of the components of the metabolic syndrome (MetS) and four inflammatory markers, namely high sensitivity C-reactive protein (hs-CRP), the erythrocyte sedimentation rate, the concentration of fibrinogen and the white blood cell count.

**Methods:**

We have analyzed data collected between September 2002 and June 2009 in the Tel-Aviv medical center inflammation survey (TAMCIS). We recruited both apparently healthy individuals and individuals presenting with atherothrombotic risk factors. All participants were enrolled during their routine annual health check-up and gave their written informed consent. This is a cross sectional study in which we have fitted linear regression models using inflammatory markers as the dependant variables and adjust them according to the different components of the MetS and multiple other confounders.

**Results:**

Included were 12,072 individuals of whom there were 7,760 men at a mean (S.D.) age of 44 (11) years, and 4,312 women aged 44 (11) years. A significant correlation was noted between most components of the MetS and all inflammatory markers, the most significant one being with hs-CRP. In the multi-adjusted regression analysis, waist was the factor that best explained the variability of hs-CRP, in both women and men. It also remained a significant variable for the other inflammatory markers.

**Conclusions:**

From amongst the various components of the MetS, waist circumference appears to exert the most influence upon the presence and intensity of the micro-inflammatory response.

## Background

The metabolic syndrome (MetS) is associated with the presence of a low grade sub-clinical inflammatory process, so called micro-inflammation [1-7]. The relationship between this process and the risk of insulin resistance development, a hallmark of the MetS,[7-9] as well as the risk of cardiovascular morbidity and mortality,[10-12] has been previously described. Therefore, it was suggested that the detection and quantification of micro-inflammation in patients with the MetS might be of clinical relevance [13]. Whilst most studies have used the highly sensitive C-reactive protein (hs-CRP) assay for the detection and quantification of micro-inflammation other commonly used and established markers might be also relevant. These include the Westergren erythrocyte sedimentation rate (ESR),[14] the white blood cell count (WBCC),[15] and quantitative fibrinogen concentrations [16].

In order to evaluate the contribution of the MetS components (elevated waist circumference, low high-density lipoprotein, high triglycerides, impaired fasting glucose and elevated blood pressure) to the micro-inflammatory process, this cross sectional study has analyzed the strength of the association between each MetS component and four established inflammatory markers. The relative influence of the components of the MetS on these inflammatory markers may be of clinical significance aiding in the establishment of clinical guidelines for health care providers as well to public health policy makers.

## Methods

### Study Population

In the present study we analyzed the data collected at the Tel-Aviv Medical Center Inflammation Survey (TAMCIS), a registered data bank of the Israeli Ministry of Justice [17-20]. This is a relatively large survey comprising of apparently healthy individuals attending a center for periodic health examinations. Subjects attending the center for a routine health examination between September 2002 and June 2009 were invited to participate in the TAMCIS. We recruited both apparently healthy individuals and individuals presenting with atherothrombotic risk factors. All the individuals who were enrolled were recruited during their routine annual health check-up and gave their written consent in accordance with the guidelines of the institutional ethics committee. A total of 15,605 subjects gave their informed consent (9,881 males, 5,724 females). Later, 2,797 subjects were excluded from the analysis due to any malignancy, immunosuppressive therapy, known inflammatory disease (arthritis, inflammatory bowel disease, psoriasis, etc.), pregnancy, steroidal or non-steroidal treatment (except for aspirin at a dose of ≤ 325 mg/day), acute infection or invasive procedures (surgery, catheterization, etc.) during the last 6 months. An additional 168 subjects were further excluded because they had no recorded hs-CRP values. The chance that diabetics harbor multiple additional inflammatory confounders such as use of statins[21] and anti-hyperglycemic medications[22-24], hidden infections[25], and yet undetermined inflammatory mechanisms[26] is high. Therefore, we have decided to narrow the scope of our analysis by excluding diabetics, including any individual taking medications for diabetes. Thus, 568 individuals were finally excluded due to a suspected or confirmed diagnosis of diabetes mellitus. Following these exclusions the study group comprised of 12,072 individuals (7,760 males and 4,312 females). These were all people visiting for the first time.

### Laboratory Methods

Blood samples were drawn in the morning hours, after a 12-h overnight fast. The WBCC and differential were performed using the Coulter STKS (Beckman Coulter, Nyon, Swiss) electronic analyzer, while fibrinogen concentrations were determined by the method of Clauss[27] and a Sysmax 6000 (Sysmex Corporation, Hyaga, Japan) analyzer. High sensitivity C-Reaeactive protein concentrations were determined by using the Behring BN II Nephelometer (DADE Behring, Marburg, Germany) analyzer and a method described according to Rifai et al. [28]. Glucose, triglycerides and high density lipoprotein cholesterol were measured using a Bayer Advia 1650 chemistry analyzer and Bayer respective kits (Bayer healthcare diagnostics division, Newbury, UK).

### Definition Of Atherothrombotic Risk Factors

The results of the routine health check-up were evaluated by employing certain definitions of the various atherothrombotic risk factors. Diabetes mellitus was defined as a fasting blood glucose level of ≥ 126 mg/dl (7 mmol/L) or treatment with insulin or oral hypoglycemic medications. Hypertension was defined as displaying with blood pressure of ≥ 140/90 mmHg in two separate measurements or the intake of anti-hypertensive medications. Dyslipidemia was defined as the low density lipoprotein cholesterol (LDL-C) or non- high density lipoprotein cholesterol (non-HDL-C) concentrations, for individuals displaying elevated triglyceride concentrations of ≥ 200 mg/dl (2.26 mmol/l) above the recommended levels, according to the risk profile defined by the updated adult treatment panel III (ATP III) recommendations[29], or the intake of lipid lowering medications. The Diagnosis of the Metabolic Syndrome was based on the National cholesterol education program ATP III Criteria [29]. The criteria for impaired fasting glucose is that used by the American Diabetes Association [30] as proposed by the updated American Heart Association/National Heart, Lung, and Blood Institute scientific statement [31]. In summary, elevated waist circumference was defined as ≥ 102 cm (40 inches) in men and ≥ 88 (35 inches) in women; Elevated triglycerides were defined as ≥ 150 mg/dl (1.7 mmol/l) or a person receiving drug treatment for elevated triglycerides; Reduced HDL-C was defined as ≤ 40 mg/dL (1.03 mmol/l) in men and ≤ 50 mg/dl (1.3 mmol/l) in women or a person receiving drug treatment for reduced HDL-C; Elevated blood pressure was defined as ≥ 130 mm Hg systolic blood pressure or ≥ 85 mm Hg diastolic blood pressure or a person receiving antihypertensive drug treatment; Elevated fasting glucose was defined as ≥ 100 mg/dl. Smokers were defined as individuals who smoked at least 5 cigarettes per day while past smokers had stopped smoking for at least 30 days prior to examination. Measured waist circumference was defined according to the National cholesterol education program's ATP III guidelines [29]. To measure the waist circumference, we located the top of the right iliac crest, placed a measuring tape in a horizontal plane around the abdomen immediately above the level of the iliac crest. Measurements were made at the end of a normal expiration.

### Statistical Methods

All data was summarized and displayed as mean ± standard deviation (SD) for the continuous variables and as number of patients plus the percentage in each group for categorical variables. Since hs-CRP, ESR and the triglyceride concentrations display irregular distributions, we used a logarithmic transformation which converted the distributions to normal ones for all statistical procedures. Therefore, all results of hs-CRP, ESR or triglyceride concentrations are expressed as back transformed geometrical means and standard deviations. The One-Way Kolmogorov-Smirnov test was used to assess the distributions. For all categorical variables the Chi-Square statistic was used to assess the statistical significance between the two genders. Pearson partial correlations for confounding variables were used to evaluate the age adjusted association between the various components of the MetS and the inflammatory variables. In order to assess and compare the contribution of the different components of the MetS to the variability of the various inflammatory variables, we used linear regression models. The inflammatory variables were the dependent variables and the different components of the MetS, as well as other potential confounders, were the covariates. The confounders included age, history of proven atherothrombotic disease (myocardial infarction, coronary artery bypass graft surgery, cerebrovascular event or peripheral artery occlusion disease), smoking status, alcohol consumption, level of physical activity and medication with potential influence on the inflammatory markers and/or the metabolic components such as angiotensin converting enzyme (ACE) inhibitors, angiotensin II receptor blockers, statins, fibrates and aspirin, as well as hormone replacement therapy or oral contraceptives in females. In an attempt to adjust for the association between the inflammatory variables (mainly hs-CRP) and obesity, we repeated the correlations and the linear regression models with additional adjustment for BMI. All above analyses were considered significant at p < 0.05 (two tailed). The statistical package for the Social Sciences (SPSS) was used to perform all statistical evaluation (SSPS Inc., Chicago, IL, USA).

## Results

We have presently analyzed a total of 7,760 men at a mean (S.D.) age of 44 (11) years (range 18-83) and 4,312 women aged 44 (11) years (range 18-77). The frequencies of the different components per each participant are described in Table 1. It can be seen that the different components of the MetS differ significantly between the two genders. The age adjusted Pearson partial coefficients of correlations between the number of MetS components and each component of MetS and between the inflammatory markers are shown in Table 2, for both genders. The hs-CRP concentrations correlated significantly with all components of the MetS in both genders. A relatively high correlation between waist and the inflammatory markers was found. The results of the regression analysis are reported in Table 3. Waist was the variable that explained most of the variability of hs-CRP, ESR and fibrinogen in both women and men. It remained significant also for the WBCC in both genders, but as the second most predominant contributing factor. We repeated our correlation and regression analyses making additional adjustments for BMI (data not shown). As expected, all the correlation coefficients were smaller compared to the previous analyses. Despite this, in most cases the results remained significant. As before, waist circumference showed the highest partial correlations in comparison to the other variables. Adjustment for BMI decreased all correlations, but the decline was more pronounced in females compared to males.

**Table 1:** Frequency of the different metabolic syndrome components among the cohort

	Men		Women		Chi-square Significance
	(N = 7,760)		(N = 4,312)		
	N	%	**N**.	%	
Waist circumference	1,490	19.2	1,037	24.0	< 0.001
HDL	901	11.6	642	14.9	< 0.001
Triglycerides	1,889	24.3	581	13.5	< 0.001
IFG	1,390	17.9	494	11.5	< 0.001
Elevated blood pressure	3,090	39.8	1,031	23.9	< 0.001
					
Zero Components	2,765	35.6	2,107	48.9	
One Component	2,511	32.4	1,194	27.7	
Two Components	1,512	19.5	590	13.7	< 0.001
Metabolic Syndrome	972	12.5	421	9.8	
(Three or more Components)					

* Criteria for the metabolic syndrome components are defined in the text.

**abbreviations: HDL = high density lipoprotein, IFG = impaired fasting glucose.

**Table 2:** Age adjusted Pearson partial correlations between components of the metabolic syndrome and the inflammatory biomarkers

Men (N = 7,760)	Number of Components	Waist	HDL	TG	Glucose	DBP	SBP
Log(hs-CRP)	0.262^§^	0.345^§^	-0.197^§^	0.191^§^	0.082^§^	0.131^§^	0.083^§^
Log(ESR)	0.080^§^	0.118^§^	-0.092^§^	0.082^§^	0.015	0.018	-0.005
Fibrinogen	0.102^§^	0.170^§^	-0.092^§^	0.063^§^	0.037^§^	0.070^§^	0.017
WBCC	0.189^§^	0.182^§^	-0.128^§^	0.216^§^	0.006	0.108^§^	0.099^§^

**Women** **(N = 4,312)**	**Number of Components**	**Waist**	**HDL**	**TG**	**Glucose**	**DBP**	**SBP**

Log(hs-CRP)	0.362^§^	0.405^§^	-0.151^§^	0.379^§^	0.143^§^	0.186^§^	0.198^§^
Log(ESR)	0.126^§^	0.169^§^	-0.090^§^	0.136^§^	0.050^§^	0.052^§^	0.049^§^
Fibrinogen	0.171^§^	0.253^§^	-0.082^§^	0.142^§^	0.037^‡^	0.106^§^	0.091^§^
WBCC	0.216^§^	0.163^§^	-0.128^§^	0.258^§^	0.043^‡^	0.096^§^	0.113^§^

* Criteria for the metabolic syndrome components are defined in the text.

**Abbreviations: hs-CRP = high sensitivity C-reactive protein; ESR = erythrocyte sedimentation rate; WBCC = white blood cell count; HDL = high density lipoprotein; TG = triglycerides; DBP = diastolic blood pressure, SBP = systolic blood pressure.

***‡ - 0.01 < P < 0.05; § - P < 0.01

**Table 3:** Metabolic syndrome components ordered according to the strength of the association to each inflammatory biomarker.

Inflammatory variable	Gender	Variable	Non-standardized Coefficients		Significance	Partial Correlation
			B	S.E.		
Log(hs-CRP)	Men	Waist	0.013	0.001	< 0.001	0.274
(R^2 ^= 0.17)		HDL	-0.004	0.001	< 0.001	-0.095
		Log(TG)	0.111	0.026	< 0.001	0.052
		DBP	0.003	0.001	0.002	0.038
		Glucose	0.001	0.001	0.045	0.024
		SBP	0.000	0.001	0.571	-0.007
						
(R^2 ^= 0.33)	Women	Waist	0.016	0.001	< 0.001	0.331
		Log(TG)	0.459	0.038	< 0.001	0.189
		HDL	-0.002	0.001	< 0.001	-0.057
		Glucose	0.002	0.001	0.062	0.030
		SBP	0.001	0.001	0.104	0.026
		DBP	0.001	0.001	0.382	0.014

Log(ESR)	Men	Waist	0.003	< 0.001	< 0.001	0.091
(R^2 ^= 0.07)		HDL	-0.002	< 0.001	< 0.001	-0.045
		Log(TG)	0.062	0.021	0.003	0.037
		SBP	< 0.001	< 0.001	0.059	-0.024
		Glucose	< 0.001	< 0.001	0.689	-0.005
		DBP	< 0.001	0.001	0.584	0.007
						
(R^2 ^= 0.07)	Women	Waist	0.003	< 0.001	< 0.001	0.114
		Log(TG)	0.105	0.024	< 0.001	0.072
		HDL	< 0.001	< 0.001	0.100	-0.027
		SBP	< 0.001	< 0.001	0.370	-0.015
		Glucose	< 0.001	0.001	0.507	-0.011
		DBP	< 0.001	0.001	0.746	0.005

Fibrinogen	Men	Waist	0.873	0.074	< 0.001	0.141
(R^2 ^= 0.14)		DBP	0.560	0.116	< 0.001	0.059
		HDL	-0.250	0.070	< 0.001	-0.043
		SBP	-0.210	0.067	0.002	-0.038
		Glucose	0.096	0.077	0.210	0.015
		Log(TG)	-3.648	3.364	0.278	-0.013
						
(R^2 ^= 0.12)	Women	Waist	1.282	0.095	< 0.001	0.214
		DBP	0.363	0.181	0.045	0.032
		Log(TG)	7.253	5.106	0.156	0.023
		Glucose	-0.157	0.110	0.153	-0.023
		HDL	-0.086	0.068	0.202	-0.021
		SBP	-0.103	0.100	0.303	-0.017

WBCC	Men	Log(TG)	1.153	0.097	< 0.001	0.146
(R^2 ^= 0.12)		Waist	0.019	0.002	< 0.001	0.109
		Glucose	-0.008	0.002	0.001	-0.042
		DBP	0.010	0.003	0.002	0.038
		SBP	0.005	0.002	0.013	0.031
		HDL	-0.004	0.002	0.047	-0.025
						
(R^2 ^= 0.13)	Women	Log(TG)	1.604	0.147	< 0.001	0.177
		Waist	0.012	0.003	< 0.001	0.071
		HDL	-0.005	0.002	0.011	-0.042
		SBP	0.007	0.003	0.014	0.041
		Glucose	-0.004	0.003	0.180	-0.022
		DBP	0.002	0.005	0.711	0.006

*Criteria for the metabolic syndrome components are defined in the text.

**All models were adjusted in addition to the different components of the MetS, to age, history of proven atherothrombotic disease, smoking status, alcohol consumption, level of physical activity and medication such as ACE inhibitors, Angiotensin II receptor blockers, statins, fibrates and aspirin, as well as hormone replacement therapy or oral contraceptives in females.

***Abbreviations: hs-CRP = high sensitivity C-reactive protein; ESR = erythrocyte sedimentation rate; WBCC = white blood cell count; HDL = high density lipoprotein; TG = triglycerides; DBP = diastolic blood pressure, SBP = systolic blood pressure.

## Discussion

The present analysis has shown that amongst the various components of the MetS, waist circumference is the component that most significantly influences the micro-inflammatory response. Invariably, Waist and BMI are used inter-changeably in the definition of the metabolic syndrome and there is in fact a strong association between them. Due to this association, adjusting for waist and BMI together causes problematic co-linearity. However, it must be noted that waist circumference still remained the most significant predictor of the inflammatory response even after additional adjustment for BMI. There is a known association between the MetS and the presence of micro-inflammation [32-34] but to the best of our knowledge, the relative contribution of the different MetS components to the low grade, sub-clinical inflammatory response has not been previously evaluated. Thus, our findings contribute original information to the literature being the largest analysis to date evaluating the association between inflammation and each individual component of the metabolic syndrome in adults. A previous analysis from our group regarding the hypertriglyceridemic waist phenotype, evaluated this phenotype and its relation to inflammation [35]. This phenotype however, although related to the metabolic syndrome, has different definitions from the metabolic syndrome. The previous work evaluated this difference and did not analyze the relative weight of each component of the metabolic syndrome. The importance of the current analysis stems from the detrimental effect that the presence of micro-inflammation has on the pathogenesis of atherothrombosis, insulin resistance, [7,9] and cardiovascular morbidity and mortality [10-12]. This study has analyzed commonly used inflammatory markers with known atherosclerotic significance. High sensitivity C-reactive protein has been shown to have deleterious effects on vascular biology,[33] white blood cells can contribute to endothelial injury[36] and fibrinogen is related to hyperviscosity, which in turn can also contribute to vascular events [37]. Thus, the presence of these markers could represent a link between the individual components of the MetS and the development of the atherosclerotic disease.

The prevalence of the MetS in our cohort was relatively low. In fact only 9.6% of women and 11.7% of men had three MetS components or more. One possible explanation for this is the fact that this study was performed in a group of relatively healthy individuals. However, this population could represent those individuals that are still not affected by the results of long standing atherosclerotic disease and might therefore benefit from preventive interventions. In addition, it should be stressed that we did not limit ourselves to individuals with an established diagnosis of MetS. We wanted to discover the relationship between the presence of micro-inflammation and changes in each individual component of the MetS, even in individuals without defined MetS. Theoretically, our findings can be used to support the recent report by Arnlov et al [38] that have demonstrated that overweight and obese individuals without MetS are as well at an increased risk for cardiovascular mortality and morbidity. It is crucial to understand that preventative measures should be implemented early, even when only one or two components are present, since even a mild increase in inflammation and the presence of one or two MetS components can be associated with a significant increase in future risk of MetS development. Our study is therefore relevant not only for those individuals who meet all the criteria of the MetS but even for individuals who are in a pre-MetS state. Furthermore, the clinical importance of our findings is also strengthened by the work of Ridker et al. [12]. The Jupiter trial showed that statin therapy in apparently healthy persons without hyperlipidemia but with elevated C-reactive protein levels significantly reduced the incidence of major cardiovascular events. Thus, elevated waist circumference as the primary contributor of the inflammatory state in the MetS, could by itself in the future be a possible indication for statin therapy [39].

In conclusion, a clarification of the relationship between each MetS component and the intensity of the micro-inflammatory response may be of clinical relevance. Such a clarification might help to highlight the importance of targeted interventions such as weight reduction, a measure previously proved to be clearly beneficial [40,41].

## Competing interests

The authors declare that they have no competing interests.

## Authors' contributions

OR and AS have participated in the design of the study, performed the statistical analyses and drafted the paper. SB and IS conceived the study, participated in its design and coordination and helped to draft and review the manuscript. TF, TC and OKB helped in the data organization and retrieval, English editing and final draft preparation. All of the authors have read and approved the final manuscript.
